# Effect of Pulsed Electric Field on Membrane Lipids and Oxidative Injury of *Salmonella typhimurium*

**DOI:** 10.3390/ijms17081374

**Published:** 2016-08-22

**Authors:** Ou Yun, Xin-An Zeng, Charles S. Brennan, Zhong Han

**Affiliations:** 1 School of Food Science and Engineering, South China University of Technology, Guangzhou 510641, China; cloud_ou@126.com (O.Y.); fezhonghan@scut.edu.cn (Z.H.); 2 Centre for Food Research and Innovation, Department of Wine, Food and Molecular Biosciences, Lincoln University, Lincoln 85084, New Zealand

**Keywords:** pulsed electric fields, *Salmonella typhimurium*, membrane lipids, cytoplasmic membrane fluidity, oxidative injury

## Abstract

*Salmonella typhimurium* cells were subjected to pulsed electric field (PEF) treatment at 25 kV/cm for 0–4 ms to investigate the effect of PEF on the cytoplasmic membrane lipids and oxidative injury of cells. Results indicated that PEF treatment induced a decrease of membrane fluidity of *Salmonella typhimurium* (*S. typhimuriumi*), possibly due to the alterations of fatty acid biosynthesis-associated gene expressions (down-regulation of *cfa* and *fabA* gene expressions and the up-regulation of *fabD* gene expression), which, in turn, modified the composition of membrane lipid (decrease in the content ratio of unsaturated fatty acids to saturated fatty acids). In addition, oxidative injury induced by PEF treatment was associated with an increase in the content of malondialdehyde. The up-regulation of cytochrome bo oxidase gene expressions (*cyoA*, *cyoB*, and *cyoC*) indicated that membrane damage was induced by PEF treatment, which was related to the repairing mechanism of alleviating the oxidative injury caused by PEF treatment. Based on these results, we achieved better understanding of microbial injury induced by PEF, suggesting that micro-organisms tend to decrease membrane fluidity in response to PEF treatment and, thus, a greater membrane fluidity might improve the efficiency of PEF treatment to inactivate micro-organisms.

## 1. Introduction

Pulsed electric field (PEF) technology has been researched extensively in the area of non-thermal pasteurization as an innovative and environmental friendly technology which causes minimal changes to the sensory quality and nutritional ingredient of liquid foods [1,2,3,4]. In addition, the combination of PEF with other hurdles, such as heat and antimicrobial compounds, has been proposed to improve the pasteurization efficiency of PEF treatment [5,6] and the saftey of semi-solid foods [7,8,9].

The mechanism by which PEF inactivates micro-organisms is mainly ascribed to electroporation [10]. The reversible pores on cytoplasmic membrane induced by PEF result in the injury of micro-organisms, while the irreversible pores on cytoplasmic membrane caused by PEF has been shown to lead to microbial death [11,12,13]. Studies have also illustrated that oxidative damage of cell may be caused by PEF treatment [14]. For instance, a previous study illustrated that an oxidative jump could be induced by PEF treatment at 0.9 kV/cm [15]. In addition, red blood cells have been shown to have suffered from oxidative injury after exposure to electric fields [16]. However, micro-organism damage induced by PEF, especially in the aspect of oxidative damage, still required more work to achieve better understanding of the mechanism by which PEF inactivates bacteria.

Cytoplasmic membrane fluidity and membrane fatty acid composition play important roles in the response of micro-organisms to external stress [17]. The key target of PEF inactivating micro-organisms is the membrane, PEF has the capability of modifying organizational changes of membrane, which ultimately affects vital activities of micro-organisms and leads to cell damage or cell death [18]. Although several studies have demonstrated that PEF treatment decreased membrane fluidity [19] and altered membrane fatty acid composition [20] of *Saccharomyces cerevisiae*, little is known about the relationship between PEF treatment and modification of membrane fatty acid composition at the transcriptional level of micro-organisms.

The objective of the present study was to investigate the effect of PEF on lipids and oxidative injury of *Salmonella typhimurium* based on the cytoplasmic membrane fluidity, fatty acid composition, and lipid peroxidation of *S. typhimurium*. In addition, the effects of PEF on the transcription of cytochrome bo oxidase genes (*cyoA*, *cyoB*, *cyoC*) [18] and fatty acid biosynthesis-associated genes (*cfa*, *fabA*, *fabD*) [21] were determined to identify stress behavior of *S. typhimurium* in response to PEF and obtain more information on microbial inactivation by PEF.

## 2. Results

### 2.1. Salmonella typhimurium (S. typhimurium) Inactivation by Pulsed Electric Field (PEF) Treatment

**Figure 1:**
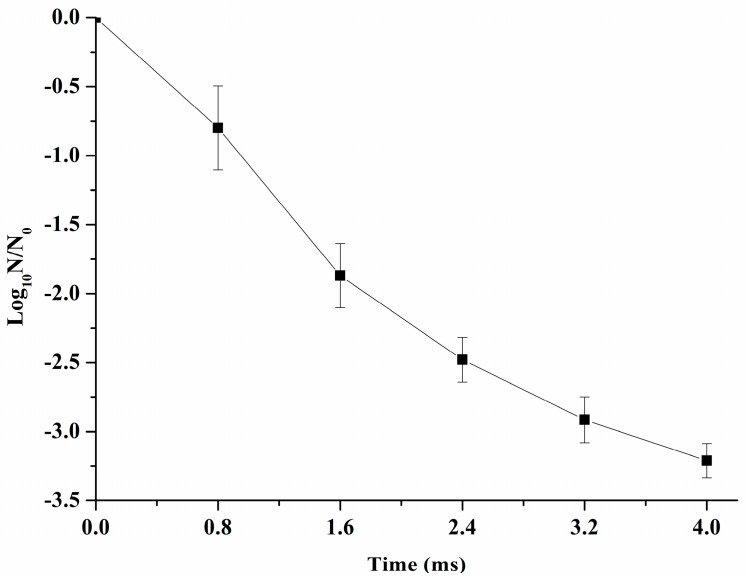
The inactivation of *Salmonella typhimurium* (*S. typhimuriumi*) by pulsed electric field (PEF) treatment at 25 kV/cm for 0–4 ms.

Figure 1 illustrates the inactivation of *S. typhimurium* by PEF treatment at 25 kV/cm with treatment time from 0–4 ms. It can be noted that with the increase of PEF treatment time from 0–4 ms the reduction of log_10_ cycles dramatically increased to 3.21 ± 0.12 (*p* < 0.05).

### 2.2. Modification in Cytoplasmic Membrane Fluidity

**Figure 2:**
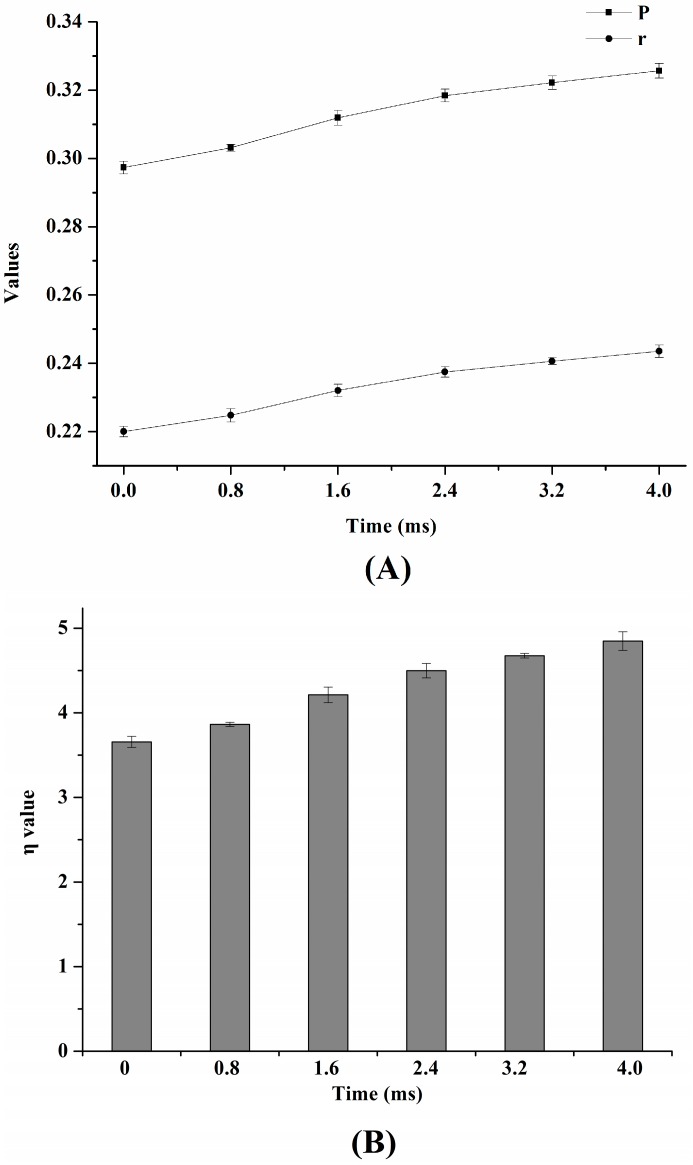
The alterations of (**A**) fluorescence polarization (*P*), fluorescence anisotropy (*r*); and (**B**) micro-viscosity (*η*) of cytoplasmic membrane of *S. typhimurium* after PEF treatments at 25 kV/cm for 0–4 ms.

1,6-diphenyl-1,3,5-hexatriene (DPH), was used as a fluorescence probe to estimate the membrane fluidity of *S. typhimurium*. Figure 2 illustrates the effects of PEF on fluorescence polarization (*P*), fluorescence anisotropy (*r*), and micro-viscosity (*η*). Compared to the control sample, *P* values of cells increased by 2.02% and 9.76% when PEF treatment times were 0.8 and 4 ms, respectively (Figure 2A). In addition, as depicted in Figure 2A, the *r* value significantly increased by 10.91% compared to the control sample after PEF treatment for 4 ms (*p* < 0.05). Similarly, the alteration of *η* values in cells increased as the PEF treatment time increased. Figure 2B illustrates a 1.32-fold increase in *η* value of cells after PEF treatments at 25 kV/cm for 4 ms compared to those without PEF treatment (*p* < 0.05), which indicates that the membrane became relatively more rigid after relatively longer PEF treatment time.

### 2.3. Changes in Lipid Composition

Table 1 shows the influence of the PEF treatment on fatty acid composition of *S. typhimurium*. Apart from nine main fatty acids shown in Table 1, three other fatty acids were also found, including C15:0 (pentadecanoic acid), C17:0 (margaric acid) and C18:0 (stearic acid). When subjected to PEF treatment for 1.6 and 4.0 ms, the content of C18:1 (octadecenoic acid) and C18:2 (octadecadienoic acid) significantly decreased and the content of C16:0 (palmitic acid) increased (*p* < 0.05). However, compared to the control sample, the contents of cyclic fatty acids after PEF treatment for 1.6 ms exhibited no significant difference (*p* > 0.05), while those after PEF treatment for 4 ms exhibited significant decrease (*p* < 0.05).

### 2.4. Lipid Peroxidation and Cell Morphology

**Figure 3:**
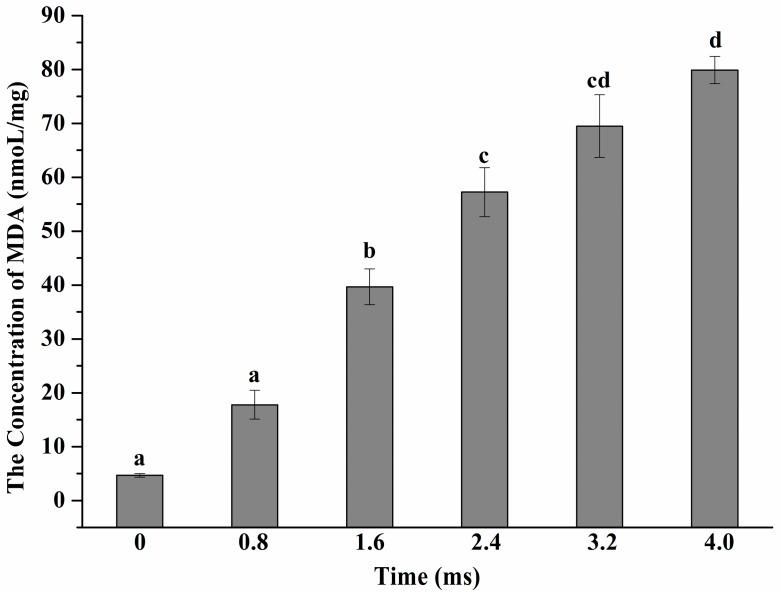
The alterations of content of malondialdehyde of *S. typhimurium* after PEF treatments at 25 kV/cm for 0–4 ms. Different letters indicate the significant difference (*p* < 0.05).

**Figure 4:**
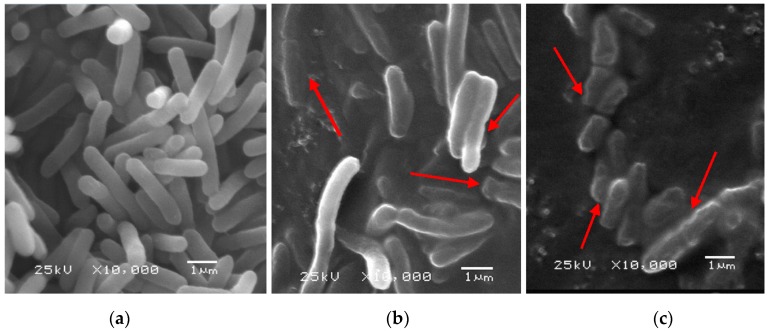
The effect of PEF treatment at 25 kV/cm with treatment time for 0, 1.6, and 4.0 ms on the morphology of *S. typhimurium*. (**a**) Control; (**b**) PEF-treated for 1.6 ms; and (**c**) PEF-treated for 4.0 ms. Arrows indicate the modifications of cell morphology and cell debris.

Figure 3 illustrates the content of malondialdehyde (MDA) generated after PEF with different treatment time. The results show an increased trend of the content of MDA with the increased PEF treatment time. The content of MDA was 79.90 ± 2.52 nmol/mg after PEF exposure for 4 ms. When the treatment time reached 1.6 ms, the content of MDA exhibited a significant difference compared to the control sample (*p* < 0.05). In addition, and consistent with the results previously described, scanning electron microscope (SEM) images illustrated that with the increment of PEF treatment time *S. typhimurium* cells suffered from severe cell injury (Figure 4). It can be revealed from SEM images that the modifications of cell morphology and cell debris had taken place after cells subjected to PEF treatment.

### 2.5. Alteration in Expression of Fatty Acid Biosynthesis-Associated Genes and Cytochrome Bo Oxidase Genes

**Figure 5:**
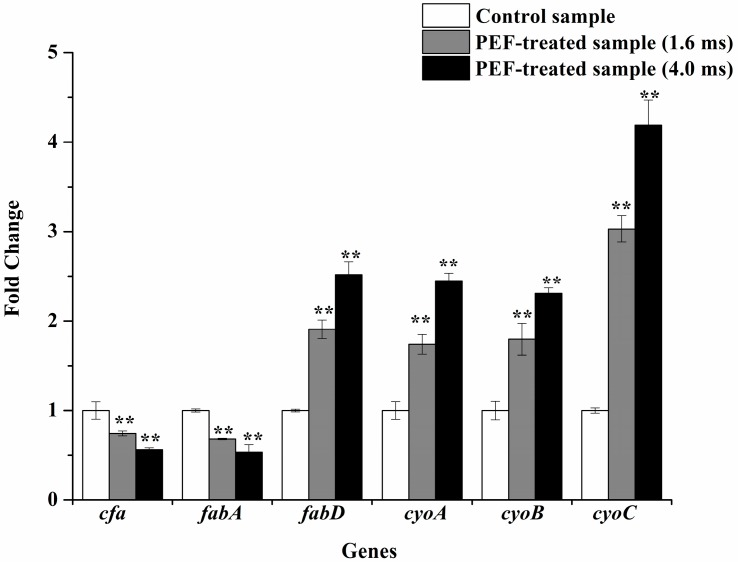
The effect of PEF treatment at 25 kV/cm on the expression of *cfa*, *fabA*, *fabD*, *cyoA*, *cyoB*, and *cyoC* genes of *S. typhimurium* (** *p* < 0.01).

The transcription levels of fatty acid biosynthesis-associated genes (*cfa*, *fabA*, and *fabD*) were determined to investigate membrane fatty acid composition of *S. typhimurium* at the transcriptional level. The results showed that compared to the control sample, the down-regulation of the expression of *cfa* and *fabA* genes were in very significant difference, while the transcription of *fabD* was very significantly up-regulated (*p* < 0.01). For instance, a 1.91-fold up-regulation of *fabD*, a 1.47-fold down-regulation of *fabA* and a 1.35-fold down-regulation of *cfa* were observed when cells were subjected to PEF treatment for 1.6 ms, respectively (Figure 5). The expression of cytochrome bo oxidase genes (*cyoA*, *cyoB*, and *cyoC*) were also determined before and after PEF treatment. All cytochrome bo oxidase genes exhibited very significant up-regulation after PEF treatment by comparison with the control sample (*p* < 0.01). For instance, the expression of *cyoC* gene after PEF treatment for 1.6 ms was more than triple of that without PEF treatment (Figure 5).

## 3. Discussion

### 3.1. The Effect of PEF Treatment Time on S. typhimurium Inactivation

With an increase of PEF treatment time, the reduction of log_10_ cycles was also increased. Compared to previous research, there was less treatment time required to inactivate three log_10_ cycles of *S. typhimurium* under similar PEF treatment conditions [4]. This might be due to the low conductivity (0.18 ms/cm) used in this study which may, in turn, influence the specific energy delivered to the sample. In addition, previous study illustrated that the specific energy input was 188 kJ/L, the *Escherichia coli* ATCC 26 of inactivation by PEF was from 1.5–3.6 log_10_ cycles when the pH of the samples ranged from 6–7 [22], which was similar to our results.

### 3.2. The Effect of PEF on Cytoplasmic Membrane Fluidity of S. typhimurium

In this paper, the results of cytoplasmic membrane fluidity modified by PEF were similar to previous studies [19,20], which also demonstrated that PEF treatment decreased membrane fluidity. Previous research illustrated that dipalmitoyl phosphatidylcholine (DPPC) and soya phosphatidylcholine (PC) vesicles subjected to heat-assisted PEF treatment exhibited greater electro-permeability than those subjected to PEF treatment [23], which was due to a greater membrane fluidity that the vesicles possessed when the temperature of thermal treatment reached the phase transition temperature of the vesicles. Remarkably, heat-assisted PEF treatment enhanced the efficiency of microbial inactivation in various liquid foods [6,18]. Thus, it is probable that a lower membrane fluidity is required to protect cells from the damage caused by PEF treatment. In addition, the occurrence of disorder in cell metabolism or cell death may result from the organizational changes of membrane induced by PEF treatment [18]. Therefore, it is also possible that PEF treatment can induce the modification in fatty acid composition of membrane. Previous research has illustrated that the alteration in content of unsaturated fatty acids (UFA) and saturated fatty acids (SFA) results in changes of the UFA to SFA ratio, which is regaded as the predominant factor affecting membrane fluidity [24].

### 3.3. The Effect of PEF on on Lipid Composition and Oxidative Injury

Lipid composition plays an important role in cytoplasmic membrane integrity and function [25]. The alterations in lipid composition of membrane are induced by bacterial adaptation to external stress [16,26], resulting in modifications in membrane integrity [27]. In this paper, all identified membrane fatty acids of *S. typhimurium* were polar lipids, which could be affected by PEF treatment [28]. The occurrence of a significant decrease in the ratio of UFA to SFA after PEF treatment was predominantly ascribed to an increase of the C16:0 and a decrease of the C18:1. These results were similar to the results of Zhao et al. (2014) [21], which indicated that PEF treatment decreased the content of UFA and increase the content of SFA of membrane in *S. cerevisiae*. A relatively lower ratio of UFA to SFA represented a relatively lower membrane fluidity [29], which was consistent with the results of modifications of fluorescence anisotropy and micro-viscosity of membrane induced by PEF. It is probable that the regulation of fatty acid desaturases is stimulated after PEF exposure, which could modulate membrane lipids composition [30]. Additionally, it is also possible that the oxidative injury induced by PEF generates free radicals that could react with fatty acids [31], which results in the decrease in the degree of unsaturation and cell damage.

MDA was regarded as an indicator of oxidative damage mainly due to the lipid peroxidation reaction between polyunsaturated fatty acid (PUFA) and the free radicals [31]. Therefore, the occurrence of a significant decrease of C18:2 in membrane lipids after PEF exposure may be ascribed to the fact that *S. typhimurium* cells had insufficient ability to adapt to PEF treatment, contributing to oxidative injury or cell death. Similarly, previous research has illustrated that oxidative injury was induced by electrostatic field in red blood cells [16]. It has been reported that membrane damage could be induced by PEF treatment and contribute to disrupting cell structure and function as well as leakage of intracellular substances [32]. Therefore, it is possible that membrane repair may be triggered when *S. typhimurium* cells are exposed to PEF treatment, which may result in the up-regulation of expression of genes related to membrane function.

### 3.4. The Effect of PEF on Relative Expression of Fatty Acid Biosynthesis-Associated Genes and Cytochrome Bo Oxidase Genes

*FabA* regulates the key fatty acid desaturase and the synthesis of UFA derived from SFA [33,34]. *FabD* encodes long-chain-fatty-acid–CoA ligase and involved in the regulation of the processes of fatty acid biosynthesis [35]. *Cfa* controls the cyclopropane fatty acyl phospholipid syntheses and participates in the biosynthesis of CFA [36]. Thus, the down-regulation of *fabA* induced by PEF treatment might imply a decrease in the biosynthesis of UFA [21]. A down-regulation of *cfa* induced by PEF might indicate a decrease in the formation of CFA. These events may be related to the decrease in the degree of unsaturation. It was interesting to mention that compared to the control sample, the contents of cyclic fatty acids after PEF treatment for 1.6 ms exhibited no significant difference (*p* > 0.05), while those after PEF treatment for 4 ms exhibited significant decrease (*p* < 0.05). It was possible that the formation of CFA was not just determined by *cfa*. For instance, Kim et al. (2005) have reported that the *rpoS* gene plays an important role in the formation of CFA of *S. typhimurium* [36]. In addition, these results were consistent with the results of membrane fluidity and lipid composition caused by PEF treatment. Therefore, PEF treatment has a tremendous impact on the expression of fatty acid biosynthesis-associated genes, which leads to the decrease in UFA to SFA ratio and membrane fluidity for adaptation of micro-organisms to PEF treatment.

The cytochrome bo oxidase is regarded as one of the predominant respiratory cytochrome oxidases on the membrane and plays an important role in generating the proton motive force [37]. The cytochrome bo oxidase genes (*cyoA*, *cyoB*, and *cyoC*) were significantly up-regulated after PEF treatment, which indicated that a proper response related to membrane function was activated by PEF treatment [9,38]. These results were in agreement of the view that membrane was the predominant target for microbial inactivation by PEF [39]. Previous reports have shown that damaged *E. coli* cells required the involvement of phospholipids synthesis and energy production to recover from membrane damage induced by PEF [40]. Additionally, the repair of membrane damages in *Listeria monocytogenes* cells was intimately related to energy production [41]. It is possible, therefore, that cell repair may be triggered after PEF treatment. Calderon et al. [38] illustrated that the cytochrome bo oxidase genes of *S. typhimurium* cells were up-regulated when subjected to NO stress, reducing their susceptibility to oxidative damage. Therefore, it is possible that *S. typhimurium* cells tended to alleviate oxidative injury caused by PEF treatment by up-regulation of *cyoA*, *cyoB*, and *cyoC*.

## 4. Materials and Methods

### 4.1. Materials

*S. typhimurium* strain (ATCC 14028) was purchased from the American Type Culture Collection (Manassas, VA, USA). Tryptic soy broth (TSB), yeast extract, peptone, and agar were obtained from Guangdong Huankai Microbial Sci. and Tech. Co., Ltd. (Guangzhou, China). 1,6-Diphenyl-1,3,5-hexatriene (DPH), methanol, hexane, and methyl *tert*-butyl ether were purchased from Aladdin Chemistry Co., Ltd. (Shanghai, China), all other chemicals used were of reagent grade and obtained from Guangzhou local market.

### 4.2. Growth Condition of S. Typhimurium

With one single colony from slant culture, broth subculture was carried out in 100 mL of sterile TSB supplemented with yeast extract (0.6%, *w*/*v*) (TSBYE) and incubated at 37 °C for 10 h in an orbital shaker (200 rpm; OS-200, Hangzhou Allsheng Instruments Co., Ltd., Hangzhou, China). After incubation, 5 mL of these subcultures, containing 20% glycerol, was transferred into sterile test tubes and then stored at −80 °C. The culture of one tube was transferred into a sterile 500 mL flask containing 200 mL of TSBYE and cultivated at 37 °C with agitation (200 rpm) until the cells reached a stationary growth phase with a final concentration of 5 × 10^9^ CFU/mL.

### 4.3. PEF System and Treatment

Before PEF treatment, the pellets were obtained by centrifuging at 4000× *g* for 5 min (JW-3021HR, Anhui Jiaven Equipment Industry Co., Ltd., Hefei, China) and then adjusted to a final concentration of 10^9^ CFU/mL by re-suspension in sterile deionized water. Two mol/L sterile potassium chloride solution was used to adjust the electrical conductivity of all samples to 0.18 ms/cm.

For PEF treatment, all samples were continuously circulated in the PEF system described in previous studies [42,43] through a rotary pump (323E/D, Watson-Marlow Inc., Wilmington, MA, USA). The PEF treatment temperature was maintained at 10 ± 1 °C by a heat exchanger (DLSK-3/10, Ketai Instrument Co., Ltd., Zhenzhou, China). The parameters of PEF system and treatment were as follows: bipolar square wave; pulse frequency: 1 kHz; pulse width: 40 μs; gap between electrodes: 0.30 cm; flow volume: 0.02 mL; flow rate: 1 mL/s; electric field strength: 25 kV/cm; times of treatment cycle from 1 to 5. The PEF treatment time *t* (s) was calculated based on Equations (1) and (2):

(1) $$t=n\times N_{p}\times W_{p}$$

(2) $$N_{p}=\frac{V\times f}{F}$$

where *n*, *N_p_*, and *W_p_* represent the times of treatment cycle, the number of pulses, and the pulse duration (μs), respectively. *V*, *f*, and *F* represent the flow volume (mL), the pulse frequency (Hz), and the flow rate (mL/s), respectively.

According to Equations (1) and (2), the PEF treatment time and the number of pulses were 0.8 ms (20 pulses), 1.6 ms (40 pulses), 2.4 ms (60 pulses), 3.2 ms (80 pulses), and 4.0 ms (100 pulses), respectively. The control sample was prepared without PEF treatment. The specific PEF energy applied to the samples during one cycle was 90 kJ/L and the temperature rise was lower than 25 °C in all process. In addition, the final pH of the treatment media was 6.70 ± 0.30.

### 4.4. Enumeration of Survivors

One milliliter of control sample was diluted by 0.1% (*w*/*v*) sterile peptone solution with gradient dilution. Then, 0.1 mL of diluted control sample was immediately placed on tryptic soy agar with 0.6% yeast extract. PEF-treated cells were conducted to the same operation as the control sample. All plates were incubated at 37 °C for 24 h. After incubation, the enumeration of viable cells was determined by computing the log_10_ reduction of the PEF-treated samples compared to the control sample.

### 4.5. Measurement of Cytoplasmic Membrane Fluidity

The determination of fluorescence polarization, fluorescence anisotropy and micro-viscosity of cells was carried out according to the methods of Zhang et al. [20]. Briefly, the pellets were collected by centrifugation (4000× *g*, 5 min, 4 °C), followed by washing three times by sterile 10 mL of buffer solution, containing 0.06 mol/L disodium phosphate, 0.02 mol/L monosodium phosphate, and 0.15 mol/L sodium chloride. 4 mL of 2 μmol/L DPH solution was used to re-suspend the pellets in test tubes. Subsequently, the tubes were maintained at 37 °C for 30 min in a water bath with darkness. After centrifugation (4000× *g*, 5 min, 4 °C), the pellets were washed three times by the buffer solution and then re-suspended in 4 mL of buffer solution. Finally, the suspensions were used to detect membrane fluidity of *S. typhimurium* cells by a spectrofluorometer (F-4500, Hitachi, Japan).

The parameters of the spectrofluorometer were as follow: slit width of excitation light: 5 nm; slit width of emission light: 5 nm; excitation wavelength: 362 nm; and emission wavelength: 432 nm. The control sample was prepared without being DPH-labeled. Equations (3)–(5) were used to calculate fluorescence polarization (*P*), fluorescence anisotropy (*r*) and micro-viscosity (*η*), respectively.

(3) $$P=\frac{I_{VV}-GI_{VH}}{I_{VV}+GI_{VH}}$$

(4) $$r=\frac{I_{VV}-GI_{VH}}{I_{VV}+2GI_{VH}}$$

(5) $$\eta =\frac{2P}{0.46-P}$$

where *I_VV_* and *I_VH_* denote the fluorescence intensities of emission polarizer oriented vertically and horizontally when the excitation polarizer is determined at the vertical position, respectively. *G* denotes the grating factor.

### 4.6. Determination of Membrane Fatty Acid Composition

Based on the method of Sasser et al. [44], pellets (40 mg) were obtained by centrifugation (4000× *g*, 5min) and re-suspended in 1 mL of water: methanol (1:1 *v*/*v*) mixture solution containing 3.75 mol/L NaOH. After vortexing (7.5 ± 2.5 s), samples were pipetted into test tubes and heated at 100 ± 1 °C for 5 min. A further 25 min heat processing (100 ± 1 °C) was conducted after a vortexing (7.5 ± 2.5 s). Afterwards, the test tubes were cooled before the addition of 2 mL of HCl (6.0 mol/L):methanol (13:11, *v*/*v*) mixture solution to each tube. The tubes were then vortexed (7.5 ± 2.5 s) and placed in a water bath at 80 ± 1 °C for 10 min followed by immediate cooling. Then 1.25 mL of hexane:methyl *tert*-butyl ether (1:1, *v*/*v*) mixture solution was added into each tube, and the tubes were tumbled for 10 min. The lower phase was then removed and 3 mL of 0.3 mol/L NaOH was added to each tube. After tumbling for a further 5 min, 2/3 of the upper phase was transferred into a gas chromatography (GC) vial which was stored at −80 °C until analysis.

The membrane fatty acid composition of *S. typhimurium* was analyzed by a GC Agilent 7820A (Agilent Technologies, Wilmington, DE, USA) loaded with a capillary column HP-5 (30 m × 0.32 mm × 0.25 μm, Agilent Technologies) using pure nitrogen as carrier gas at 1 mL/min in a split mode (20:1). Under the following temperature program: the first ramp, initial temperature was increased from 150 to 170 °C at 10 °C·min^−1^ and held for 0.5 min; the second ramp, the temperature was increased to 200 at 5 °C·min^−1^ and maintained for 1 min; the third ramp, the temperature was increased to 260 at 2 °C·min^−1^ for detection. The comparison was made between the retention times of fatty acid methyl esters and known standard (Supelco 37 Component FAME Mix, Sigma, St. Louis, MO, USA). The fatty acid methyl esters were finally identified by analysis of gas chromatograph-mass spectrometer analyses (GCMS-QP2010 Ultra, SHIMADZU, Tokyo, Japan) with the same temperature program. The percentage composition of each fatty acid was calculated as the ratio of the surface area of the considered peak to that of all peaks.

### 4.7. Detection of Membrane Lipid Peroxidation

PEF-treated cells and control cells were collected by centrifugation (4000× *g*, 5 min, 4 °C). The MDA concentrations of samples were analyzed following the instructions of Malondialdehyde (MDA) Assay Kit (Nanjing Jiancheng Bioengineering Institute, Nanjing, China). Finally, the samples were detected spectrophotometer (UV-1800, Tokyo, Japan) at 532 nm to determine oxidative damage of *S. typhimurium* cells induced by PEF treatment.

### 4.8. RNA Extraction and Analysis of Gene Expression by Quantitative Real-Time RT-PCR (qRT-PCR)

The extraction of total RNA in *S. typhimurium* cells was immediately carried out according to the instructions of Qiagen RNeasy Mini Kit 74106 (Qiagen Inc., Hilden, Germany) after PEF treatment. Reverse transcription was carried out following the instructions of iScript cDNA synthesis kit (Bio-Rad Inc., Philadelphia, PA, USA) and the first strand cDNA was synthesized based on 150 ng of total RNA. Analysis of genes expression was performed by 7900 HT Sequence Detection System (ABI, Foster City, CA, USA) to investigate the effects of PEF on the transcription of membrane function genes (*cyoA*, *cyoB*, *cyoC*) and fatty acid biosynthesis-associated genes (*cfa*, *fabA*, *fabD*). The synthesis of all primers was made by Sangon Biotech Ltd. (Shanghai, China) according to the data from the NCBI database (accession number NC_003197.1), which were listed in Table 2. Gene expression was detected by 10 μL of qPCR reaction mix (ABI Power SYBR Green PCR Master Mix, (ABI, Foster City, CA, USA) according to the protocol of manufacturer. The reaction mix was composed of 5 μL of 2× qPCR Master Mix, 0.5 of 10 μM forward primer, 0.5 of 10 μM reverse primer, 1 ng of template cDNA, and PCR water used to maintain the total volume to 10 μL. Control sample was prepared by reactions without reverse transcriptase. The reaction conditions were as follows: 1 cycle at 50 °C maintained for 2 min, one cycle at 95 °C held for 10 min, 40 cycles at 95 °C for 15 s and 60 °C for 1 min. 16S rRNA of *S. typhimurium* was regarded as the endogenous control. Fold changes of these gene expression were computed based on the method of 2^−ΔΔ*C*t^ [45].

### 4.9. Scanning Electron Microscope (SEM)

The morphological changes of *S. typhimurium* were observed by scanning electron microscope (JSM-6360LV, Tokyo, Japan). The PEF-treated and without PEF treatment suspensions were performed to centrifugation (4000× *g*, 5 min) and the pellets were re-suspended with paraformaldehyde:glutaraldehyde (2%:2.5%, *w*/*w*) mixture for 12 h at 4 °C. Subsequently, 0.1 mol/L phosphate solution (pH 7.2) was used to wash the samples for three times, followed by 1% osmium tetroxide fixing for 2.5 h at 25 °C. After being washed by the same buffer for three times, samples were dehydrated by graded ethanol from 30%–100% and substituted by *tert*-butyl alcohol. Then, samples were stored at −20 °C for 1 h and dried by vacuum freeze drier (SCIENTZ-18N, Shanghai, China) for 24 h. Finally, all samples were transferred into a sputter coater (JEOLJFC-1600, Tokyo, Japan) for gold sputtering and then observed by SEM.

### 4.10. Statistics Analysis

Mean values were computed based on the three replicate measurements of duplicate experiments. All data were analyzed with least significant difference (LSD) (*p* < 0.05 or *p* < 0.01) based on analysis of variance (ANOVA) by SPSS software (Statistical Package for the Social Sciences, version 22.0, IBM, Armonk, NY, USA). All data were expressed as mean ± standard deviation.

## 5. Conclusions

The present study investigated the effect of PEF on cytoplasmic membrane lipids of *S. typhimurium* by focusing on the membrane fluidity, fatty acid composition, and lipid peroxidation. Results indicate that PEF appears to alter the membrane function and cause damage to cells, contributing to decrease in membrane fluidity and oxidative injury. *S. typhimurium*, which was subjected to PEF treatment for 1.6 ms, showed a decrease in membrane fluidity. The micro-viscosity of *S. typhimurium* was increased significantly compared to the untreated cells, largely due to the significant down-regulation of *fabA* genes and up-regulation of *fabD*, which was characterized by the in alterations in composition of membrane lipid (decrease in ratio of UFA to SFA). In addition, PEF treatment resulted in oxidative injury of *S. typhimurium*, which was characterized by an increase in the content of MDA and a decrease in the content of PUFA. Exposure to PEF treatment for 1.6 and 4.0 ms, resulted in a significant up-regulation the expression of *cyoA*, *cyoB*, and *cyoC* genes. This might mitigate the oxidative injury, suggesting that *S. typhimurium* might tend to a decreased membrane fluidity in response to PEF and a greater membrane fluidity, which could improve the efficiency of PEF treatment to inactivate micro-organisms. More attention should be focused on the effect of PEF on membrane function regulation of micro-organisms to reveal the microbial response behavior to PEF treatment.

## Figures and Tables

**Table 1:** Fatty acids composition of stationary phase cells of *Salmonella typhimurium* (*S. typhimuriumi*) before and after pulsed electric field (PEF) treatment at 25 kV/cm.

Fatty Acid Composition	Content (%)		
	Control	PEF for 1.6 ms	PEF for 4.0 ms
Saturated fatty acid (SFA)			
C12:0	2.11 ± 0.32 ^a^	1.96 ± 0.15 ^a^	1.65 ± 0.12 ^b^
C14:0	6.71 ± 0.48 ^a^	5.35 ± 0.12 ^ab^	5.81 ± 0.31 ^a^
C16:0	46.04 ± 1.23 ^a^	50.90 ± 0.23 ^b^	55.68 ± 0.62 ^c^
Unsaturated fatty acid (UFA)			
C16:1	5.56 ± 0.41 ^a^	5.06 ± 0.98 ^a^	4.84 ± 0.37 ^a^
C18:1	7.30 ± 0.12 ^a^	4.54 ± 0.21 ^b^	3.01 ± 0.16 ^c^
Polyunsaturated fatty acid (PUFA)			
C18:2	1.33 ± 0.11 ^a^	0.63 ± 0.09 ^b^	0.22 ± 0.08 ^c^
Cyclic fatty acid (CFA)			
C17:cyclo	19.21 ± 0.37 ^a^	18.91 ± 0.93 ^a^	16.22 ± 0.21 ^b^
C19:cyclo	3.23 ± 0.62 ^a^	2.94 ± 0.21 ^a^	1.75 ± 0.14 ^b^
C14:0 (3-OH)	7.40 ± 0.12 ^a^	7.76 ± 0.15 ^a^	8.13 ± 0.16 ^ab^
Total minor fatty acids	1.11 ± 0.59 ^a^	1.95 ± 0.49 ^a^	2.69 ± 0.68 ^ab^

Different letters indicate the significant difference (*p* < 0.05).

**Table 2:** Primers of fatty acid biosynthesis-associated genes and cytochrome bo oxidase genes used in this study.

Gene	Sequence (5′ to 3′)	Product Length (bp)
16S rRNA	F: TCGTGTTGTGAAATGTTGGGTTA	66
	R: ACCGCTGGCAACAAAGGAT	
*fabA*	F: GGTTCTTCGGATGCCACTTTAT	65
	R: CATAGCATCCAGACCCAGACAA	
*fabD*	F: AGTGGACGAAGAGCGTGGAAT	67
	R: CCTGGACCCACTTCATAAAGATG	
*cfa*	F: CCCCCACCATGTTAAAGATACG	74
	R: AGGCGCGTTTTTTACTTTGTAGA	
*cyoA*	F: TGGTTTCGCCTGGAAGTATC	64
	R: GTGTGACCAGTTCGGGCTAT	
*cyoB*	F: GGCACCCATTTCTTTACCAA	105
	R: GACCGGCAGAATCAGAATGT	
*cyoC*	F: GGATGGCGGTGCTGATG	67
	R: ATGATGCGGGTACGGTTAGTG	
